# A plea to stop using the case‐control design in retrospective database studies

**DOI:** 10.1002/sim.8215

**Published:** 2019-08-22

**Authors:** Martijn J. Schuemie, Patrick B. Ryan, Kenneth K.C. Man, Ian C.K. Wong, Marc A. Suchard, George Hripcsak

**Affiliations:** ^1^ Observational Health Data Sciences and Informatics New York New York; ^2^ Epidemiology Analytics Janssen Research and Development Titusville New Jersey; ^3^ Department of Biostatistics University of California Los Angeles California; ^4^ Department of Biomedical Informatics Columbia University Medical Center New York New York; ^5^ Centre for Safe Medication Practice and Research, Department of Pharmacology and Pharmacy Li Ka Shing Faculty of Medicine, The University of Hong Kong Pokfulam Hong Kong; ^6^ Research Department of Practice and Policy UCL School of Pharmacy London UK; ^7^ Department of Medical Informatics Erasmus University Medical Center Rotterdam The Netherlands; ^8^ Department of Social Work and Social Administration, Faculty of Social Science The University of Hong Kong Pokfulam Hong Kong; ^9^ Department of Biomathematics University of California Los Angeles California; ^10^ Department of Human Genetics University of California Los Angeles California; ^11^ Medical Informatics Services NewYork‐Presbyterian Hospital New York New York

**Keywords:** case control, database studies, methods, retrospective studies

## Abstract

The case‐control design is widely used in retrospective database studies, often leading to spectacular findings. However, results of these studies often cannot be replicated, and the advantage of this design over others is questionable. To demonstrate the shortcomings of applications of this design, we replicate two published case‐control studies. The first investigates isotretinoin and ulcerative colitis using a simple case‐control design. The second focuses on dipeptidyl peptidase‐4 inhibitors and acute pancreatitis, using a nested case‐control design. We include large sets of negative control exposures (where the true odds ratio is believed to be 1) in both studies. Both replication studies produce effect size estimates consistent with the original studies, but also generate estimates for the negative control exposures showing substantial residual bias. In contrast, applying a self‐controlled design to answer the same questions using the same data reveals far less bias. Although the case‐control design in general is not at fault, its application in retrospective database studies, where all exposure and covariate data for the entire cohort are available, is unnecessary, as other alternatives such as cohort and self‐controlled designs are available. Moreover, by focusing on cases and controls it opens the door to inappropriate comparisons between exposure groups, leading to confounding for which the design has few options to adjust for. We argue that this design should no longer be used in these types of data. At the very least, negative control exposures should be used to prove that the concerns raised here do not apply.

## 1 BACKGROUND

Case‐control^1^ studies consider the question “are persons with a specific disease outcome exposed more frequently to a specific agent than those without the disease?” Thus, the central idea is to compare “cases,” ie, individuals that experience the outcome of interest with “controls,” ie, individuals that did not experience the outcome of interest. Often, one matches controls to cases based on characteristics such as age and sex to make them more comparable. The comparison focuses on differential exposure to the agents of interest in the two groups; greater exposure amongst the cases than amongst the controls suggests a possible positive association. Perhaps the greatest success of the case‐control design stands its contribution to the evidence that smoking causes lung cancer.^2^ Since this landmark finding, researchers increasingly have applied the design, occasionally generating spectacular findings leading to headlines in major news outlets, such as a recent study linking anticholinergic drugs to an increased risk of dementia,^3^ or another recent study linking immunosuppressants to a lower risk of Parkinson's disease.^4^

The case‐control design was originally developed to support prospective studies in situations where data on subjects were costly to acquire, and study budgets did not allow for recruiting and following large cohorts.^5^ However, its application in retrospective database studies, for example those using electronic health records and insurance claims where longitudinal person‐level data have already been captured and the analysis is performed solely within the resident data, has become commonplace. Because the data already exist, and their acquisition is a sunk cost, the efficiency argument that initially justified the design becomes a moot point. The added value over other designs for these type of retrospective data is therefore questionable. Along these lines, Schneeweiss provided similar conceptual argument against this design in 2010^6^; our prior research published in 2013 showed empirical evidence that it is prone to substantial bias when using retrospective data,^7^ and others have reported on the lack of reproducibility of such case‐control studies in 1988.^8^ Yet, despite all these prior concerns, the number of publications applying this design has increased dramatically over time. Using a specific PubMed query, we identify 1801 publications in the last 5 years that employ the case‐control design in a retrospective database study (see Supplementary Materials).

Here, we aim to describe the mechanisms that lead to bias in clinical case‐control studies using retrospective longitudinal data. We reproduce two published studies to demonstrate this problem, showing that both studies are likely severely biased, and show that an alternative design is equally viable given the same data, and demonstrates considerably less bias. We not only argue against further use of the case‐control designs in situations where other designs such as a comparative cohort analysis or self‐controlled design are equally viable, but we also highlight a set of study diagnostics that researcher should perform if they insist on using a case‐control design anyway.

### 1.1 Issues with the case‐control design

One root cause of trouble is that the case‐control design focuses on the definition of cases and their controls, while the main research question centers on exposed and unexposed. Being “exposed” is often defined as the presence of some intervention in the patient's record at the time of the outcome, while “unexposed” indicates the absence of such an intervention. However, this begs the question whether exposed and unexposed are comparable. Is someone who has received a treatment for some serious ailment really comparable to someone else who did not receive that treatment, even when matching on age and sex, or nesting in a subpopulation with a particular disease? While other designs such as the new‐user cohort design makes this comparison explicit, the case‐control design obfuscates this essential question of comparability.

A related issue stems from the fact that the case‐control design is anchored on the date of the outcome. Any covariates that we may capture to adjust for differences relative to this date might actually be captured *after* the exposure of interest was started, thus opening the possibility of erroneously adjusting for causal intermediaries. This problem becomes uncomfortably clear if we try to rephrase a case‐control study as the equivalent cohort study. New‐user cohort studies are anchored on the date the exposure starts, thus allowing baseline covariates to be captured before exposure and ruling out causal intermediaries. However, often the unexposed group implied by a case‐control study has no well‐defined index date when follow‐up starts.

One final argument against using a case‐control design when the data is already available to also perform a cohort study is efficiency, ie, by using only a sample of the controls information on other controls is discarded, and a case‐control study estimate will therefore always have lower precision than the estimate of an equivalent cohort study using all data.

**Methods**

We replicate two recent studies that represent a range of approaches taken in case‐control studies. The first study, by Crockett et al,^9^ investigates the effect of isotretinoin on the risk of several outcomes including ulcerative colitis (UC) using a fairly simple design. The second study, by Chou et al,^10^ investigates dipeptidyl peptidase‐4 (DPP‐4) inhibitors on the risk of acute pancreatitis, employing a more complex design with nesting in a cohort of type‐2 diabetes mellitus patients, and additional confounding adjustment through covariates included in a multivariable regression. We replicate these two studies as faithfully as possible, and additionally include a set of negative control exposures that are not believed to cause the outcomes of interest such that their true odds ratios (ORs) should be equal to 1. Applying the same design used in the replication studies to these controls allows us to quantify residual bias in the design.^11^, ^12^

An overview of our methods is provided next. The full protocol, attached as supplementary information, provides specific details such as the codes used to identify disease conditions and drug exposures, and the list of negative control outcomes. The computer code for executing our study is available as open source software at https://github.com/OHDSI/StudyProtocols/tree/master/EvaluatingCaseControl.

### 1.2 Data source

For our analyses, we use the IBM® MarketScan® Commercial Claims and Encounters Database (CCAE) that represent longitudinal data from individuals enrolled in United States employer‐sponsored insurance health plans. The data include adjudicated health insurance claims (eg, inpatient, outpatient, and outpatient pharmacy) as well as enrollment data from large employers and health plans that provide private healthcare coverage to employees, their spouses, and dependents. Additionally, CCAE captures laboratory tests for a subset of the covered lives. This administrative claims database includes a variety of fee‐for‐service, preferred provider organizations, and capitated health plans, captured from March 2000 up to and including September 2017. The data are transformed to the OMOP Common Data Model version 5.1.

In contrast, the Crockett study used the PharMetrics Patient‐Centric Database (IMS Health, Watertown, MA), another US insurance claims database. The Chou study used the Taiwanese National Health Insurance Research Database.

### 1.3 Crockett study replication

Both the original study by Crockett et al and our replication define cases as subjects having at least three health‐care contacts with a UC diagnosis code on three different dates, or one UC diagnosis code and exposure to a drug used in UC treatment. Controls are selected from the overall population, matching on age (with 2‐year caliper), gender, and length of enrollment (90‐day caliper). The index date is defined as the date of the outcome for cases, and the date exactly 12 months after enrollment start for controls. The exposure of interest is isotretinoin. Subjects are considered “exposed” if they are exposed any time in the 12 months prior to the index date. One noteworthy difference with the original study is that our replication does not match on region or health plan since those data are not available in the CCAE database.

#### 1.3.1 Chou study replication

Both the original study by Chou et al and our replication only consider cases and controls from a patient cohort who had at least one outpatient or inpatient diagnosis of type‐2 diabetes mellitus and who fill at least one prescription of oral antihyperglycemic agents. The cohort entry date is defined as the prescribing date of the first claim of oral antihyperglycemic agents. To be eligible for the study cohort, patients need to be 18 years old and have claims data for a continuous period of at least 12 months before the cohort entry date and 6 months after the cohort entry date. Cases are defined as subjects having an inpatient diagnosis of acute pancreatitis, with the index date set to the date of diagnosis. Up to four controls are selected by matching on age (within a 1‐year caliper), gender, and time in cohort (within a 1‐year caliper), with the index date set to the index date of the case to which the controls are matched. Subjects are considered exposed if they are exposed any time in the 30 days prior to the index date.

Additionally, the following risk factors of acute pancreatitis are included in the conditional logistic regression outcome model, based on occurrence in the year before the index date: gallstone disease, alcohol‐related disease, hypertriglyceridemia, cystic fibrosis, neoplasm, obesity, and tobacco use. Both studies also adjust for the Diabetes Complications Severity Index to account for the potential impacts of the severity of diabetes on the risk of acute pancreatitis, and include exposure to drugs that might be associated with acute pancreatitis (furosemide, NSAIDs, corticosteroids, antibiotics, and cancer drugs).

#### 1.3.2 Negative control exposures

We identify a large set of negative control exposures where we are confident they are not causally associated with the outcome of interest, such that we can assume the true OR is 1. First, we generate a candidate list of negative control exposures by identifying exposures with no evidence of being causally related to the outcome of interest.^13^ We search for this evidence in the literature through MeSH headings^14^ and natural language processing,^15^ spontaneous reports of adverse events,^16^ and product labels in the US^17^ and Europe.^18^ We then reverse sort the candidate exposures by prevalence in the longitudinal database and manually curate until at least 35 negative controls remain. For the nested case‐control study (the Chou et al replication), we define a nesting cohort for each negative control exposure by selecting one of the primary indications of each drug. The negative controls are listed in Appendix 1 of the protocol.

#### 1.3.3 Alternative study design

To demonstrate that alternative designs with better means to adjust for confounding are equally viable in retrospective database studies, we estimate the same effects using the self‐controlled case series (SCCS) design.^19^ The SCCS compares the rate of outcomes during exposure to the rate of outcomes during all unexposed time, both before, between, and after exposures. It is a Poisson regression that is conditioned on the person. Thus, it seeks to answer the following question: “Given that a patient has the outcome, is the outcome more likely during exposed time compared to nonexposed time?” Because the SCCS is a self‐controlled design, where each person serves as their own control, it is immune to confounding due to factors that are constant over time.

Crockett et al defined cases and control to be exposed if they were exposed in the 365 days prior to the index date. Therefore, when approximating that study using the SCCS design, we define subjects to be exposed starting on the day after treatment initiation and stopping 365 days after the end of their last prescription, allowing for a 30‐day gap between prescriptions. When approximating the Chou study, we define subjects to be exposed starting on the day after treatment initiation and stopping 30 days after the end of their last prescription, also allowing for a 30‐day gap between prescriptions. For both studies, we exclude the first 365 days of observation to establish exposure status at the start of follow‐up, add a pre‐exposure window of 30 days to counter any time‐varying effects due to contra‐indications, and adjust for age and season by assuming a constant effect of age and season within each calendar month and using five‐knot cubic splines to model the effect across months.

## 2 RESULTS

In the Crockett replication, we identify 122 192 cases of UC, which are matched to 366 576 controls (original study: 4428 cases and 21 832 controls). In the Chou replication, we identify 6799 cases of acute pancreatitis, which are matched to 27 196 controls (original study: 1957 cases and 7828 controls).

### 2.1 Comparability of exposed to unexposed

Table 1 reports the percentage of the 1049 exposed and 487 719 unexposed in our Crockett replication that have an occurrence of select diagnosis and medication codes in the year up to a month prior to the exposure start (for exposed) or index date (for unexposed). We exclude the last month to minimize the risk of detecting precursors of the outcome. Table 1 also reports the standardized difference of means (S.Diff) for each characteristic. Typically, an absolute S.Diff >0.1 is considered imbalance between the exposure groups. Many characteristics exceed this threshold.

**Table 1:** Patient characteristics of exposed and unexposed in the Crockett replication. S.Diff: Standardized difference of means (exposed – unexposed)

**Characteristic**	**Exposed** %	**Unexposed** %	**S.Diff**	**Characteristic**	**Exposed** %	**Unexposed** %	**S.Diff**
Medical history: General				Peripheral vascular disease	0.6	2.4	0.11
Acute respiratory disease	16.6	21.9	0.09	Pulmonary embolism	0.0	0.2	0.04
ADHD	2.3	1.1	−0.06	Venous thrombosis	0.4	0.8	0.04
Chronic liver disease	0.6	0.9	0.03	Medical history: Neoplasms			
Chronic obstructive lung disease	0.2	1.2	0.08	Hematologic neoplasm	0.2	0.4	0.02
Crohn's disease	2.3	3.5	0.05	Malignant lymphoma	0.0	0.2	0.04
Dementia	0.2	0.1	−0.02	Malignant neoplasm of anorectum	0.0	0.1	0.03
Depressive disorder	4.2	5.6	0.04	Malignant neoplastic disease	1.0	3.2	0.11
Diabetes mellitus	0.7	5.8	0.20	Malignant tumor of breast	0.1	0.7	0.07
Gastroesophageal reflux disease	1.9	4.9	0.11	Malignant tumor of colon	0.0	0.2	0.04
Gastrointestinal hemorrhage	1.6	4.0	0.10	Malignant tumor of lung	0.0	0.1	0.03
HIV infection	0.3	0.2	−0.02	Malignant tumor of urinary bladder	0.0	0.1	0.03
Hyperlipidemia	3.3	16.0	0.29	Prostate cancer	0.0	0.3	0.05
Hypertensive disorder	2.4	15.5	0.31	Medication use			
Lesion of liver	0.1	0.3	0.03	Agents acting on the RAS	1.6	11.6	0.27
Obesity	0.5	2.4	0.11	Antibacterials for systemic use	58.9	43.4	−0.15
Osteoarthritis	1.9	6.8	0.17	Antidepressants	9.5	14.9	0.11
Pneumonia	0.8	1.2	0.03	Antiepileptics	2.8	5.3	0.09
Psoriasis	0.9	0.8	−0.01	Antiinflam. and antirheum. drugs	8.7	16.0	0.15
Renal impairment	0.2	0.8	0.06	Antineoplastic agents	14.9	2.2	−0.31
Rheumatoid arthritis	0.3	0.8	0.05	Antipsoriatics	5.5	0.5	−0.21
Schizophrenia	0.0	0.1	0.02	Antithrombotic agents	0.8	2.5	0.10
Ulcerative colitis	0.0	0.0	0.02	Beta blocking agents	1.6	7.2	0.19
Urinary tract infectious disease	3.0	5.2	0.08	Calcium channel blockers	1.0	4.7	0.16
Viral hepatitis C	0.0	0.3	0.05	Diuretics	3.8	9.7	0.16
Visual system disorder	6.6	11.9	0.12	Drugs for acid related disorders	5.7	12.2	0.15
Medical history: Cardiovascular disease							
Atrial fibrillation	0.1	0.6	0.06	Drugs used in diabetes	0.7	4.8	0.18
Cerebrovascular disease	0.3	0.7	0.04	Immunosuppressants	2.3	3.0	0.03
Coronary arteriosclerosis	0.1	1.9	0.13	Lipid modifying agents	1.5	12.4	0.29
Heart disease	1.5	6.4	0.17	Opioids	5.8	11.9	0.14
Heart failure	0.3	0.5	0.03	Psycholeptics	7.7	14.6	0.15
Ischemic heart disease	0.1	1.2	0.10	Psychostimulants and nootropics	3.3	2.7	−0.02

Table 2 reports the same statistics for the 4933 exposed and 29 062 unexposed in the Chou replication. One characteristic exceeds our predefined threshold, and the S.Diff is positive for almost all, suggesting those not exposed to DPP‐4 inhibitors are overall sicker than those that are not exposed.

**Table 2:** Patient characteristics of exposed and unexposed in the Chou replication

**Characteristic**	**Exposed** %	**Unexposed** %	**S.Diff**	**Characteristic**	**Exposed** %	**Unexposed** %	**S.Diff**
Medical history: General				Peripheral vascular disease	6.4	7.9	0.04
Acute respiratory disease	24.8	24.0	−0.01	Pulmonary embolism	0.2	0.4	0.02
ADHD	0.4	0.5	0.01	Venous thrombosis	1.4	1.6	0.02
Chronic liver disease	3.7	3.4	−0.01	Medical history: Neoplasms			
Chronic obstructive lung disease	2.4	3.6	0.05	Hematologic neoplasm	0.6	0.9	0.02
Crohn's disease	0.4	0.4	0.00	Malignant lymphoma	0.3	0.4	0.01
Dementia	0.1	0.2	0.01	Malignant neoplasm of anorectum	0.2	0.1	0.00
Depressive disorder	5.7	8.0	0.06	Malignant neoplastic disease	4.5	5.6	0.04
Diabetes mellitus	78.2	85.2	0.05	Malignant tumor of breast	0.9	1.0	0.01
Gastroesophageal reflux disease	7.8	8.3	0.01	Malignant tumor of colon	0.3	0.3	0.01
Gastrointestinal hemorrhage	2.3	2.4	0.00	Malignant tumor of lung	0.1	0.2	0.03
HIV infection	0.4	0.2	−0.02	Malignant tumor of urinary bladder	0.0	0.2	0.03
Hyperlipidemia	46.2	50.4	0.04	Prostate cancer	0.5	0.8	0.02
Hypertensive disorder	49.8	54.8	0.05	Medication use			
Lesion of liver	0.6	1.2	0.04	Agents acting on the RAS	53.1	57.6	0.04
Obesity	7.5	11.8	0.10	Antibacterials for systemic use	53.7	53.5	0.00
Osteoarthritis	12.3	14.6	0.05	Antidepressants	20.8	23.5	0.04
Pneumonia	1.9	2.6	0.03	Antiepileptics	10.0	11.8	0.04
Psoriasis	1.1	1.1	0.00	Antiinflam. and antirheum. drugs	25.7	27.1	0.02
Renal impairment	3.6	4.3	0.03	Antineoplastic agents	1.5	2.0	0.03
Rheumatoid arthritis	0.9	1.2	0.02	Antipsoriatics	0.5	0.6	0.01
Schizophrenia	0.1	0.2	0.02	Antithrombotic agents	7.9	9.9	0.05
Ulcerative colitis	0.3	0.4	0.01	Beta blocking agents	23.4	25.9	0.04
Urinary tract infectious disease	6.1	7.2	0.03	Calcium channel blockers	16.3	18.7	0.04
Viral hepatitis C	0.8	0.7	0.00	Diuretics	33.5	36.1	0.03
Visual system disorder	18.5	21.6	0.05	Drugs for acid related disorders	21.8	23.1	0.02
Medical history: Cardiovascular disease				Drugs for obstructive airway diseases	20.7	21.9	−0.06
Atrial fibrillation	1.5	2.2	0.04	Drugs used in diabetes	64.2	86.1	0.18
Cerebrovascular disease	1.4	2.1	0.03	Immunosuppressants	2.0	2.5	0.02
Coronary arteriosclerosis	7.5	8.6	0.03	Lipid modifying agents	50.8	55.3	0.04
Heart disease	16.7	18.6	0.03	Opioids	19.9	20.8	0.01
Heart failure	2.5	2.9	0.02	Psycholeptics	19.9	22.6	0.04
Ischemic heart disease	4.3	4.9	0.02	Psychostimulants and nootropics	2.6	2.6	0.00

### 2.2 Case‐control effect size estimates and residual bias

The left panel of Figure 2 evaluates the OR estimate and its standard error for isotretinoin exposure both in the original study by Crockett et al (OR = 4.36, 95% confidence interval (CI) = 1.97 to 9.66), and our replication study (OR = 3.51, CI = 3.11 to 3.96) . The figure also includes the negative control exposure estimates, executed under the same study design. The full list of negative controls and their estimates can be found in the Supplementary Table S1. Note that, even though for the negative controls the true odds ratio is assumed to be 1, almost all have an estimated OR that would be called significantly (*p* < 0.05) greater than 1.

**Figure 1:**
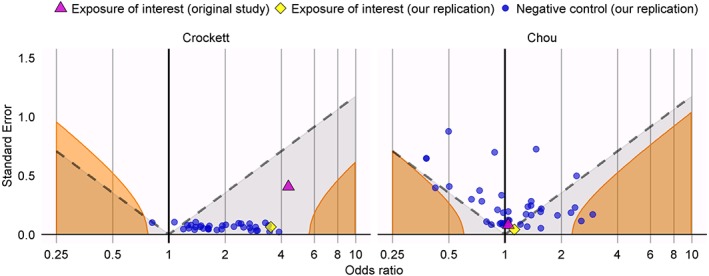
Estimates from the original study (purple triangle), our replication (yellow diamond), and our replication applied to the negative control exposures (blue dots) for the Crockett replication (left) and the Chou replication (right). Estimates below the dashed line have p < 0.05. Estimates in the orange area have a calibrated p < 0.05 [Colour figure can be viewed at wileyonlinelibrary.com]

The right panel of Figure 1 evaluates the OR estimate and its standard error for DPP‐4 inhibitor exposure both in the original study by Chou et al (OR = 1.04; CI = 0.89 to 1.21), and our replication study (OR = 1.12, CI = 1.03 to 1.22). This figure also includes the negative control exposure estimates, executed under the same study design. Many negative controls show spurious statistically significant associations. Furthermore, the fact that the orange boundary (which indicates where the calibrated p‐value^12^ is equal to 0.05), deviates strongly from the dashed line (indicating where the uncalibrated p‐value is equal to 0.05) demonstrates that overall the variability of the estimates of the negative controls is greater than can be explained by random error alone. While our replication study yields a statistically significant effect under a nominal p‐value, which does not account for systematic error, this finding is unlikely to inform us about the true effect size, but rather reflects the range of estimates one would expect to observe using this design even when no true effect exists.

### 2.3 Self‐controlled case series effect size estimates and residual bias

**Figure 2:**
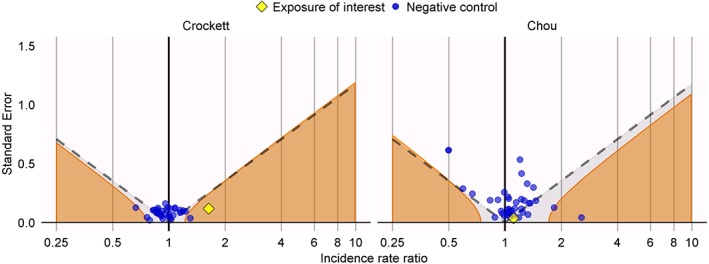
Estimates from the self‐controlled case series (SCCS) design for the exposures of interest (yellow diamond) and the negative control exposures (blue dots) for the Crockett replication (left) and the Chou replication (right). Estimates below the dashed line have p < 0.05. Estimates in the orange area have a calibrated p < 0.05 [Colour figure can be viewed at wileyonlinelibrary.com]

The left panel of Figure 2 shows the incidence rate ratio (IRR) estimates generated using the SCCS for the question addressed in the Crockett study as well as the negative controls. A much smaller effect (IRR = 1.64; CI = 1.30 to 2.06) is observed for isotretinoin than in both the original case‐control study and our replication, but in the SCCS analysis, this effect clearly stands out compared to the negative controls, suggesting there might be a true effect. The right panel of Figure 2 shows the effect estimates for DPP‐4 inhibitors (IRR = 1.11; CI = 1.04 to 1.19) are similar to the case‐control studies. Even though the negative controls show less bias than when using the case‐control design, this estimate would still be consistent with the effects observed for the negative controls. The full list of SCCS estimates can be found in the Supplementary Table S2.

## 3 DISCUSSION

Our replications of the two published case‐control studies produce similar effect size estimates to the original studies, with highly overlapping CIs, but we also show that these findings are likely caused by bias. This explains why others studying the same questions have found different answers. For example, Bernstein et al^20^ reported no association between isotretinoin and UC (OR = 1.16, CI = 0.56 to 2.20), and Singh et al^21^ reported a positive association between sitagliptin (a DPP‐4 inhibitor) and acute pancreatitis (OR = 2.01, CI = 1.19 to 3.38). Our use of negative controls clearly shows that these case‐control designs tend to produce biased estimates. We hypothesize that this bias is due to confounding that stems from differences between the exposure groups and show that exposed and unexposed indeed have different baseline characteristics.

This brings us to our main criticism of applying the case‐control design when other designs are viable, ie, by focusing on cases and controls, this design hides the question of comparability between exposed and unexposed. As a consequence, applications of the case‐control design often imply inappropriate comparisons between exposure groups, where especially the unexposed group is often ill‐defined. This, in turn, leads to confounding, and because the design anchors on the outcome date rather than the exposure date, our hands are tied when we try to adjust for this confounding, because we must avoid including causal intermediaries.

We have attempted to reframe the Crockett and Chou studies as comparative new‐user cohort studies, but found it impossible to identify appropriate comparator groups with index dates. For example, when should follow‐up start for people not exposed to isotretinoin in the cohort‐study equivalent of the Crockett study? Rather than being a limitation of the cohort design, we believe these difficulties reveal the inappropriateness of the comparison implied by these case‐control designs.

As an alternative, we reframed the studies as SCCS^19^ instead. Because of the self‐controlled nature of the SCCS design, it addresses the type of confounding observed for the case‐control studies, and may often be suitable for use in these type of data.^22^ Consequently, our analysis of the same data using the SCCS designs yields considerably less bias as observed through the negative control estimates.

A typical response to the biases reported here is that these findings do not apply to any given researcher's specific case‐control study; people agree that the two example studies we show here are problematic, but if we had only modified the designs in a specific way, all the issues we observe would go away. For example, the use of propensity scores^23^ or disease risk scores^24^ could address between person confounding in case‐control studies, but our concern with the use of these scores is the danger of accidentally including intermediary variables, as well as other issues reported earlier.^25^ In general, we find it hard to justify such a blind faith in the ability of minor tweaks to remedy the weaknesses inherent to the case‐control design for these types of data and argue that empirical evidence is needed to support such claims. Moreover, it is important to remember that the examples we chose reflect what is actually used in published case‐control studies today.

## 4 CONCLUSIONS

We cannot stress enough that our criticism does not apply to the case‐control design in general. In theory, even a case‐control design applied in a retrospective database study may well be unbiased if all issues raised in this paper are addressed. However, our main point is that, in such database studies, where all exposure and covariate data are available for the entire cohort, it is unnecessary to conduct a case‐control study, as other alternatives such as the cohort and SCCS designs are available. Outside of such database studies, a case‐control design may be completely justified to reduce the cost of acquiring the necessary data.

In this paper, we have highlighted several diagnostics that can reveal issues with a case‐control study design. If researchers insist on using the case‐control design, we believe the onus lies on them to use these diagnostics to verify that the concerns raised here do not apply to their study.

## Supporting information

SIM_8215‐Supp‐0001‐ProtocolRevisionV02.pdf

SIM_8215‐Supp‐0002PublicationCountV02.pdf

SIM_8215‐Supp‐0003‐SupplementaryTableS1.csv

SIM_8215‐Supp‐0004SupplementaryTableS2.csv
