# Supplementary material for: A plea to stop using the case‐control design in retrospective database studies
Source: Stat Med. 2019 Aug 22;38(22):4199–208. doi: 10.1002/sim.8215 (PMC6771795; doi:10.1002/sim.8215)
Supplement: Supplementary file 1 — SIM_8215‐Supp‐0001‐ProtocolRevisionV02.pdf [file SIM-38-4199-s001.pdf]

Evaluating the Case-Control Design Study

Version: 2.0

Authors:

Martijn Schuemie, Janssen Research and Development

Patrick Ryan, Janssen Research and Development

Kenneth Man, University of Hong Kong

Ian Wong, University of Hong Kong

Marc Suchard, University of California, Los Angeles

George Hripcsack, Columbia University

Date: 28 February 2019

Acknowledgment: The analysis is based in part on work from the Observational Health Sciences and Informatics collaborative. OHDSI (http://ohdsi.org) is a multi-stakeholder, interdisciplinary collaborative to create open-source solutions that bring out the value of observational health data through large-scale analytics.

The authors declare the following disclosures: Dr. Schuemie and Ryan are employees of Janssen Research & Development.

1 Table of contents

1 Table of contents 2
2 List of abbreviations 3
3 Abstract 3
4 Amendments and Updates 3
5 Milestones 4
6 Rationale and Background 4
7 Study Objectives 4
7.1 Primary Hypotheses 4
7.2 Secondary Hypotheses 5
7.3 Primary Objectives 5
7.4 Secondary Objectives 5
8 Research methods 5
8.1 Study Design 5
8.2 Data Source(s) 6
8.3 Study population 6
8.3.1 Replication of Crockett study (ulcerative colitis) 6
8.3.2 Replication of Chou study (acute pancreatitis) 6
8.4 Exposures 8
8.4.1 Isotretinoin 8
8.4.2 DPP-4 inhibitors 8
8.4.3 Negative control exposures 9
8.4.4 Positive control exposures 11
8.5 Outcomes 11
8.5.1 Ulcerative colitis 11
8.5.2 Acute pancreatitis 12
8.6 Covariates 13
8.6.1 Replication of Crockett study (ulcerative colitis) 13
8.6.2 Replication of Chou study (acute pancreatitis) 13
8.6.3 SCCS design 15
9 Data Analysis Plan 15
9.1 Calculation of time-at-risk 15
9.1.1 Replication of Crockett study (ulcerative colitis) 15
9.1.2 Replication of Chou study (acute pancreatitis) 15
9.1.3 SCCS approximation of the Crockett study (ulcerative colitis) 15
9.1.4 SCCS approximation of the Chou study (acute pancreatitis) 15
9.2 Model Specification 15
9.2.1 Replication of Crockett study (ulcerative colitis) 15
9.2.2 Replication of Chou study (acute pancreatitis) 16
9.2.3 SCCS approximation of the Crockett study (ulcerative colitis) 16
9.2.4 SCCS approximation of the Chou study (acute pancreatitis) 16
9.2.5 Pooling effect estimates across databases 16
9.3 Analyses to perform 16
9.4 Output 17
9.5 Evidence Evaluation 17
10 Study Diagnostics 17
10.1 Sample Size and Study Power 17

11 Strengths and Limitations of the Research Methods....17
12 Protection of Human Subjects....18
13 Management and Reporting of Adverse Events and Adverse Reactions....18
14 Plans for Disseminating and Communicating Study Results....18
15 Appendix 1....18
15.1 Negative control exposures for the Crockett study replication....18
15.2 Negative control exposures for the Chou study replication....19
16 References....20

2 List of abbreviations

ATC	Anatomic Therapeutic Chemical
CI	Confidence Interval
DCSI	Diabetes Complications Severity Index
DPP-4	Dipeptidyl peptidase-4
ICD-9	International Classification of Diseases, Ninth edition
MDRR	Minimum detectable relative risk
O	Outcome cohort
OHDSI	Observational Health Data Sciences and Informatics
OMOP	Observational Medical Outcomes Partnership
PS	Propensity Scores
SNOMED	Systematized Nomenclature of Medicine
T	Target cohort
T2DM	Type-2 diabetes mellites

3 Abstract

This study aims to evaluate the case-control study design. We will replicate two published case-control studies, one investigating the effect of isotretinoin on the risk of ulcerative colitis, and one investigating dipeptidyl peptidase-4 inhibitors on the risk of acute pancreatitis. We will include negative control exposures to quantify bias inherent in both studies, and we will generate diagnostics to explore the reasons for any observed bias.

4 Amendments and Updates

0.1	27 March 2018	M.Schuemie	First draft
0.2	30 March 2018	M. Schuemie, P. Ryan	Added confidence interval calibration
1.0	27 June 2018	M. Schuemie, P. Ryan, K. Man, I. Wong, M. Suchard, G. Hripcsack	Final, approved version
2.0	28 February 2019	M. Schuemie, P. Ryan, K. Man, I.	Added SCCS analyses. Changed characterization to focus on comparison of exposed to unexposed.

		Wong, M.<br>Suchard, G.<br>Hripcsack	

5 Milestones

Milestone	Planned / Estimated Date
Start of analysis	March 2018
End of analysis	April 2018
Submission of manuscript	June 2018

6 Rationale and Background

Case–control [1] studies consider the question ‘‘are persons with a specific condition exposed more frequently to a specific drug than those without the disease?’’ Thus, the central idea is to compare ‘‘cases,’’ i.e., individuals that experience the outcome of interest with (possibly) matched ‘‘controls,’’ i.e., individuals that did not experience the outcome of interest. The comparison focuses on differential exposure to the drug of interest in the two groups; greater exposure amongst the cases than amongst the controls suggests a possible positive association. The case–control design was developed in situations where data on subjects was costly to acquire, and study budgets did not allow for recruiting and following large cohorts [2]. Nowadays, case–control studies are typically nested in a cohort, such as the population in a longitudinal observational database, where there is little cost in retrieving data on more subjects, and where therefore the added value over other designs is questionable. Furthermore, the OMOP experiment showed the case-control design to be prone to substantial bias [3]. Despite these concerns, the case-control design remains popular. Here we aim to evaluate the case-control design by replicating two recently published studies, and investigate performance to determine whether further use of the case-control design by the observational research community is justified.

The first study [4] investigates the effect of isotretinoin on the risk of ulcerative colitis using a fairly simple design. The second study [5] investigating dipeptidyl peptidase-4 (DPP-4) inhibitors on the risk of acute pancreatitis, employing a more complex design with nesting in a cohort of type-2 diabetes mellites (T2DM) patients and additional confounding adjustment through covariates included in a multivariable regression. We attempt to replicate these two studies as faithfully as possible, and additionally include a set of negative control exposures that are not believed to cause the outcomes of interest, and where therefore the true odds ratio should equal 1. Applying the same design used in the replication studies to these controls will allow us to quantify any residual bias. Additionally, we will investigate confounding by comparing baseline characteristics of the exposed and unexposed. As an alternative to the case-control design, we will apply a self-controlled design to answer the same questions.

7 Study Objectives

7.1 Primary Hypotheses

This study’s primary hypotheses are:

- Applying the design used in the two studies to the set of negative controls will lead to much larger deviation in estimates from the truth than expected based on random error.

7.2 Secondary Hypotheses

- Despite matching and nesting, exposed and unexposed will differ in several baseline characteristics.
- Applying a self-controlled design to the set of negative controls will show a much smaller deviation in estimates from the truth than expected based on random error, compared to the case-control design.

7.3 Primary Objectives

- To estimate the risk of **O: ulcerative colitis** in **T: users of isotretinoin**, and a set of negative control exposures.
- To estimate the risk of **O: acute pancreatitis** in **T: users of DPP-4 inhibitors**, and a set of negative control exposures.

7.4 Secondary Objectives

- To report select baseline characteristics of exposed and unexposed in both studies.
- To estimate the same risk as mentioned in the primary objective, using a self-controlled design instead of the case-control design.

8 Research methods

8.1 Study Design

Both replications will follow a retrospective, observational, case-control design. We define 'retrospective' to mean the study will be conducted using data already collected prior to the start of the study. We define 'observational' to mean there is no intervention or treatment assignment imposed by the study. We define 'case' to mean a person who experiences an outcome of interest, and the 'index date' to refer to the time the outcome occurs. We define 'control' to mean a person who did not experience the outcome of interest. Cases are typically matched to control, and the index date of a control is often defined to be the index date of the matched case. We define 'case-control design' to mean the formal comparison between the group of cases and the group of controls, for the occurrence of an exposure during a defined time prior to the index date [6]. A 'nested case-control design' is taken to mean a case-control design where both cases and controls are selected from a cohort of people who share some defined criteria.

In addition to the case-control designs, we will also generate estimates for the same questions using a Self-Controlled Case Series (SCCS) design. [7]

The designs will be conducted in one administrative claims database in the US, as described in section 8.2. The specific exposure cohorts are described in section 8.3 and 8.4. The time-at-risk definitions are described in section 9.1. The statistical analysis plan for population-level effect estimation is described in

section 9.2.

8.2 Data Source(s)

The analyses will be performed across one observational database. This database has been transformed into the OMOP Common Data Model, version 5.1. The complete specification for OMOP Common Data Model, version 5.1 is available at: https://github.com/OHDSI/CommonDataModel.

Data sources expected to participate to include:

- Truven Health MarketScan® Commercial Claims and Encounters Database

This database is described below:

- Truven Health MarketScan® Commercial Claims and Encounters Database

Truven Health MarketScan® Commercial Claims and Encounters Database (CCAE) represent data from individuals enrolled in United States employer-sponsored insurance health plans. The data includes adjudicated health insurance claims (e.g. inpatient, outpatient, and outpatient pharmacy) as well as enrollment data from large employers and health plans who provide private healthcare coverage to employees, their spouses, and dependents. Additionally, it captures laboratory tests for a subset of the covered lives. This administrative claims database includes a variety of fee-for-service, preferred provider organizations, and capitated health plans.

The major data elements contained within this database are outpatient pharmacy dispensing claims (coded with National Drug Codes (NDC), inpatient and outpatient medical claims which provide procedure codes (coded in CPT-4, HCPCs, ICD-9-CM or ICD-10-PCS) and diagnosis codes (coded in ICD-9-CM or ICD-10-CM). The data also contain selected laboratory test results (those sent to a contracted thirds-party laboratory service provider) for a non-random sample of the population (coded with LOINC codes).

For this study, we will use the version of this database referred to as v698 (internally at JnJ), which spans March 2000 up to and including September 2017.

8.3 Study population

8.3.1 Replication of Crockett study (ulcerative colitis)

In the Crockett study, cases and controls were selected from the entire population captured in the database. The database used in this study was the PharMetrics Patient-Centric Database (IMS Health, Watertown, MA).

In our replication, we will similarly select cases and controls from the entire population, in our case from the CCAE database.

8.3.2 Replication of Chou study (acute pancreatitis)

The Chou study was nested in a cohort of patients with T2DM:

"We identified a type 2 diabetic patient cohort who had at least one outpatient or inpatient diagnosis of type 2 diabetes [International Classification of Diseases, Ninth edition, Clinical Modification (ICD-9-CM) code of 250.xx] and who filled at least one prescription of oral antihyperglycemic agents between 1 January 2001 and 31 December 2011. The cohort entry date was defined as the prescribing date of the first claim of oral antihyperglycemic agents. To be eligible for the study cohort, patients needed to be 18 years old and had claims data for a continuous period of at least 12 months before the cohort entry date and 6 months after the cohort entry date." [5]

We used the following cohort definition:

Initial Event Cohort

People having any of the following:

- a drug exposure of Antihyperglycemics[1]
    - with age >= 18

with continuous observation of at least 365 days prior and 180 days after event index date (entry date), and limit initial events to: **earliest event per person.**

For people matching the Primary Events, include:
Having all of the following criteria:

- at least 1 occurrences of a condition occurrence of Type 2 Diabetes Mellitus[2] starting between all days Before and 0 days After event index date (entry date)

Limit cohort of initial events to: **earliest event per person.**
Limit qualifying cohort to: **all events per person.**

End Date Strategy

No end date strategy selected. By default, the cohort end date will be the end of the observation period that contains the index event.

Cohort Collapse Strategy:

Collapse cohort by era with a gap size of 0 days.

Appendix 1: Concept Set Definitions

1. Antihyperglycemics

Concept Id	Concept Name	Domain	Vocabulary	Excluded	Descendants	Mapped
21600744	BLOOD GLUCOSE LOWERING DRUGS, EXCL. INSULINS	Drug	ATC	NO	YES	NO

2. Type 2 Diabetes Mellitus

Concept Id	Concept Name	Domain	Vocabulary	Excluded	Descendants	Mapped
201826	Type 2 diabetes mellitus	Condition	SNOMED	NO	YES	NO

8.4 Exposures

8.4.1 Isotretinoin

Initial Event Cohort

People having any of the following:

- a drug era of Isotretinoin[1]

with continuous observation of at least 0 days prior and 0 days after event index date (entry date), and limit initial events to: **all events per person.**

Limit qualifying cohort to: **all events per person.**

End Date Strategy

No end date strategy selected. By default, the cohort end date will be the end of the observation period that contains the index event.

Cohort Collapse Strategy:

Collapse cohort by era with a gap size of 0 days.

Appendix 1: Concept Set Definitions

1. Isotretinoin

Concept Id	Concept Name	Domain	Vocabulary	Excluded	Descendants	Mapped
984232	Isotretinoin	Drug	RxNorm	NO	NO	NO

8.4.2 DPP-4 inhibitors

"The DPP-4 inhibitors included in this study were sitagliptin, saxagliptin, and vildagliptin. " [5]

DPP4 users Chou replication

Initial Event Cohort

People having any of the following:

- a drug era of DPP-4 inhibitors[1]

with continuous observation of at least 0 days prior and 0 days after event index date (entry date), and limit initial events to: **all events per person.**

Limit qualifying cohort to: **all events per person.**

End Date Strategy

No end date strategy selected. By default, the cohort end date will be the end of the observation period that contains the index event.

Cohort Collapse Strategy:

Collapse cohort by era with a gap size of 0 days.

Appendix 1: Concept Set Definitions

1. DPP-4 inhibitors

Concept Id	Concept Name	Domain	Vocabulary	Excluded	Descendants	Mapped
40166035	saxagliptin	Drug	RxNorm	NO	YES	NO
1580747	sitagliptin	Drug	RxNorm	NO	YES	NO
19122137	vildagliptin	Drug	RxNorm	NO	YES	NO

8.4.3 Negative control exposures

Negative controls are concepts known to not be causally associated with the outcome of interest, such that we can assume the true odds ratio is 1. Negative controls are selected using a similar process to that outlined by Voss et al. [8]. Person counts of all potential drug-condition pairs are reviewed in observational data; this person count data helps determine which pairs are even probable for use in calibration. Given the list of potential drug-condition pairs, the concepts in the pairs must meet the following requirements to be considered as negative controls: (1) that there is no Medline abstract where the MeSH terms suggest an association between the drug and the condition [9], (2) that there is no mention of the drug-condition pair on a US Product Label in the "Adverse Drug Reactions" or "Postmarketing" section [10], (3) there are no US spontaneous reports suggesting that the pair is in an adverse event relationship [11, 12], (4) that the OMOP Vocabulary does not suggest that the drug is indicated for the condition, (5) that the concepts are usable (i.e. not too broad, not suggestive of an adverse event relationship, not pregnancy related), and (6) the exact concept itself is utilized in patient level data (i.e. concepts that are not usually used within the data are usually indicative a broad concept that has a child that is more specific). The remaining concepts are "optimized", meaning parent concepts remove children as defined by the OMOP Vocabulary (e.g. if both "Non-Hodgkin's Lymphoma" and "B-Cell Lymphoma" we selected, child concept "B-Cell Lymphoma would be removed for its parent "Non-Hodgkin's Lymphoma"). Once potential negative control candidates were selected, manual clinical review to exclude any pairs that may still be in a causal relationship or similar to the study outcome was be performed to select the top concepts by patient

exposure. The final list can be found in appendices 15.1 and 15.2.

For each negative control exposure, a patient enters the negative control exposure cohort when prescribed one of the ingredients identified in the list, and exists the exposure cohort at end of exposure, allowing for a 30-day gap between subsequent prescriptions.

For the nested case-control study (the Chou et al. replication), a nesting cohort is defined for each negative control exposure. Subjects enter a nesting cohort at the first occurrence of the nesting concept ID or any of its descendants, and remains in the nesting cohort until end of observation.

8.4.4 Positive control exposures

In addition to negative control exposures, we will also include synthetic positive control exposures. These are exposures based on the real negative controls, but where the true effect size is artificially increased to a desired effect size by injection of additional, simulated outcomes [13]. To preserve confounding, these additional outcomes are sampled from predicted probabilities generated using a fitted predictive model. For each negative control target exposure, three positive control exposures will be generated with true relative risk is 1.5, 2, and 4.

8.5 Outcomes

8.5.1 Ulcerative colitis

"subjects with at least three health-care contacts, on different days, associated with an ICD-9-CM diagnosis code for … UC (556.xx), or subjects with at least one claim for … UC, and at least one pharmacy claim for any of the following medications: mesalamine, olsalazine, balsalazide, sulfasalazine, 6-mercaptopurine, azathioprine, infl iximab, adalimumab, and enteral budesonide." [4]

Initial Event Cohort

People having any of the following:

- a condition occurrence of Ulcerative colitis[2]

with continuous observation of at least 0 days prior and 0 days after event index date (entry date), and limit initial events to: **earliest event per person.**

For people matching the Primary Events, include:
Having any of the following criteria:

- at least 3 occurrences of a condition occurrence of Ulcerative colitis[2]

  starting between 0 days Before and all days After event index date (entry date)

- or at least 1 occurrences of a drug exposure of Inflammatory bower disease medications[1]

  starting between all days Before and all days After event index date (entry date)

Limit cohort of initial events to: **earliest event per person.**
Limit qualifying cohort to: **all events per person.**

End Date Strategy

No end date strategy selected. By default, the cohort end date will be the end of the observation period that contains the index event.

Cohort Collapse Strategy:

Collapse cohort by era with a gap size of 0 days.

Appendix 1: Concept Set Definitions

1. Inflammatory bower disease medications

Concept Id	Concept Name	Domain	Vocabulary	Excluded	Descendants	Mapped
1119119	adalimumab	Drug	RxNorm	NO	YES	NO
19014878	Azathioprine	Drug	RxNorm	NO	YES	NO
934262	balsalazide	Drug	RxNorm	NO	YES	NO
939259	Budesonide	Drug	RxNorm	NO	YES	NO
937368	infliximab	Drug	RxNorm	NO	YES	NO
1436650	mercaptopurine	Drug	RxNorm	NO	YES	NO
968426	mesalamine	Drug	RxNorm	NO	YES	NO
916282	olsalazine	Drug	RxNorm	NO	YES	NO
964339	Sulfasalazine	Drug	RxNorm	NO	YES	NO

2. Ulcerative colitis

Concept Id	Concept Name	Domain	Vocabulary	Excluded	Descendants	Mapped
81893	Ulcerative colitis	Condition	SNOMED	NO	YES	NO

8.5.2 Acute pancreatitis

"We defined cases as patients who were hospitalized for acute pancreatitis during the study period (ICD-9-CM codes: 577.0)." [5]

Initial Event Cohort

People having any of the following:

- a condition occurrence of Acute pancreatitis[1]
  - visit occurrence is any of: Emergency Room Visit, Emergency Room and Inpatient Visit, Inpatient Visit

with continuous observation of at least 180 days prior and 0 days after event index date (entry date), and limit initial events to: **earliest event per person.**

Limit qualifying cohort to: **all events per person.**

End Date Strategy

No end date strategy selected. By default, the cohort end date will be the end of the observation period that contains the index event.

Cohort Collapse Strategy:

Collapse cohort by era with a gap size of 0 days.

Appendix 1: Concept Set Definitions

1. Acute pancreatitis

Concept Id	Concept Name	Domain	Vocabulary	Excluded	Descendants	Mapped
199074	Acute pancreatitis	Condition	SNOMED	NO	YES	NO

8.6 Covariates

8.6.1 Replication of Crockett study (ulcerative colitis)

"Controls were matched to cases on the following factors: age (within 2 year increments), gender, US census region (east, south, midwest, and west), health plan, and length of enrollment (in 3 month increments)." [4]

In our replication, we matched on age (with 2-year caliper), gender, and on length of observation time prior to the index date (90-day caliper). We did not match on region or health plan.

8.6.2 Replication of Chou study (acute pancreatitis)

Controls were matched on "age (±1 year), sex, and the cohort entry year." [5]

In our replication, we matched on age (within a 1-year caliper, gender, and time in cohort (within a 1-year caliper). Because we set the index date of the controls to the date of the outcome of the case they were matched to, we also match on calendar time. The Chou paper does not clearly state how the index date for the controls was selected.

"we adjusted for potential risk factors of acute pancreatitis in the statistical models. Using the outpatient

and inpatient claims of the NHIRD, we identified the following comorbidities based on data within 1 year prior to the index date: gallstone disease [ICD-9-CM codes: 560.31, 574.x], alcohol-related disease [291.x, 303.x, 305.0, 571.x (x = 03)], hypertriglyceridemia [272.1x], cystic fibrosis [277.0x], neoplasm [140.xx-209.xx], obesity [278.x (x = 0–1)], and tobacco use [305.1, 649.0, 989.84]. We also adjusted the Diabetes Complications Severity Index (DCSI) [28–30] to account for the potential impacts of the severity of diabetes on the risk of acute pancreatitis. Furthermore, we collected the exposure to drugs that might be potentially associated with acute pancreatitis within 1 year before the index date. Those drugs were furosemide, NSAIDs, corticosteroids, antibiotics, and cancer drugs." [5]

Tables 1 and list the codes used to identify the covariates in the 1 year prior to index date. Additionally, the DCSI score was computed and included.

Covariate name	ICD-9
gallstone disease	560.31
	574.??
alcohol-related disease	291.??
	303.??
	305.0
	571.0
	571.1
	571.2
	571.3
hypertriglyceridemia	272.1??
cystic fibrosis	277.0??
neoplasms	14?.??
	15?.??
	16?.??
	17?.??
	18?.??
	19?.??
	20?.??
obesity	278.0??
tobacco use	305.1
	649.0
	989.84

Table 1. ICD-9 codes used to identify covariates. Question marks (?) indicate wildcards. These codes were automatically mapped to standard concepts.

Covariate name	ATC
furosemide	C03CA01
NSAIDs	M01A
corticosteroids	H02
antibiotics	J01
cancer drugs	L01

Table 2. ATC codes used to identify covariates. All descendants of these codes were included.

8.6.3 SCCS design

In all SCCS analyses we will adjust for age and season.

9 Data Analysis Plan

9.1 Calculation of time-at-risk

Here we'll use the term 'time-at-risk' to denote the time relative to the index date when exposures are considered to be associated with the occurrence of the outcome.

9.1.1 Replication of Crockett study (ulcerative colitis)

"The number of isotretinoin prescriptions occurring in the 12 months before the first diagnosis of CD or UC for cases, or in the first 12 months of enrollment for controls …" [4]

In our replication, we considered exposure to isotretinoin in 365 days prior to the index date, which was set to the date of the outcome for cases, and 365 after observation period start for the controls.

9.1.2 Replication of Chou study (acute pancreatitis)

"The current users were those who had received DPP-4 inhibitors within 30 days before the index date …" [5]

In our replication, we considered exposure to DPP-4 inhibitors within 30 days before the index date.

9.1.3 SCCS approximation of the Crockett study (ulcerative colitis)

We define subjects to be exposed starting on the day after treatment initiation and stopping 365 days after the end of their last prescription, allowing for a 30-day gap between prescriptions.

9.1.4 SCCS approximation of the Chou study (acute pancreatitis)

We define subjects to be exposed starting on the day after treatment initiation and stopping 30 days after the end of their last prescription, also allowing for a 30-day gap between prescriptions.

9.2 Model Specification

In this study, we compared cases to controls for the occurrence of drug exposure during the time-at-risk by applying a logistic regression model.

9.2.1 Replication of Crockett study (ulcerative colitis)

Our replication of the Crockett study applies matching as an analytic strategy to reduce potential confounding due to baseline differences between cases and controls. Up to 3 controls are matched to each control on age (with 2-year caliper), gender, and on length of observation time prior to the index date (90-day caliper). The index date of cases is defined as the date of the outcome. The index date of controls is defined as 365 days after observation period start. The outcome model was conditioned on the matched sets.

9.2.2 Replication of Chou study (acute pancreatitis)

Our replication of the Chou study applies matching as an analytic strategy to reduce potential confounding due to baseline differences between cases and controls. Up to 4 controls are matched to each control on age (within a 1-year caliper, gender, and time in cohort (within a 1-year caliper). The index date of cases is defined as the date of the outcome. The index date of controls is defined as the same date as the case to which a control was matched. The outcome model was conditioned on the matched sets.

Additionally, this study includes several covariates as defined in section 8.6.2 in the logistic regression to further reduce potential confounding.

9.2.3 SCCS approximation of the Crockett study (ulcerative colitis)

We exclude the first 365 days of observation to establish exposure status at the start of follow-up, add a pre-exposure window of 30 days to counter any time-varying effects due to contra-indications, and adjust for age and season by assuming a constant effect of age and season within each calendar month and using 5-knot cubic splines to model the effect across months.

Incidence rate ratios will be estimated using a conditional Poisson regression, conditioned on the subject.

9.2.4 SCCS approximation of the Chou study (acute pancreatitis)

We exclude the first 365 days of observation to establish exposure status at the start of follow-up, add a pre-exposure window of 30 days to counter any time-varying effects due to contra-indications, and adjust for age and season by assuming a constant effect of age and season within each calendar month and using 5-knot cubic splines to model the effect across months.

Incidence rate ratios will be estimated using a conditional Poisson regression, conditioned on the subject.

9.2.5 Pooling effect estimates across databases

This study will not pool effect estimates across databases.

9.3 Analyses to perform

The following analyses will be performed:

- 2 comparisons: cases of ulcerative colitis to controls without ulcerative colitis, and cases of acute pancreatitis to controls without acute pancreatitis
- 145 exposures for ulcerative colitis: 1 exposure of interest (isotretinoin), 36 negative controls, and 36*3 = 108 positive controls.
- 157 exposures for acute pancreatitis: 1 exposure of interest (DPP-4 inhibitors), 39 negative controls, and 39*3 = 117 positive controls.
- 1 time-at-risk definition per comparison
- 2 designs per comparison (case-control and SCCS)

- 1 databases: CCAE

The total number of analyses is therefore (145 + 157) x 1 x 2 x 1 = 604 analyses.

9.4 Output

Four main results will be produced for each of the two studies:

1. A plot showing the odds ratios and standard errors for all exposures, to quantify systematic error in the designs.

2. A table showing baseline characteristics of the exposed and unexposed, and the standardized differences between them to explore overall comparability between exposed and unexposed.

3. The point estimates, confidence intervals, and p-values for the exposures of interest before and after empirical calibration, to express uncertainty due to systematic error.

9.5 Evidence Evaluation

Since the main goal of our replications is to perform study diagnostics, we only considered sample size when writing this protocol.

10 Study Diagnostics

10.1 Sample Size and Study Power

Table 3 shows the sample size and statistical power in both replication studies.

STUDY	OUTCOME	EXPOSURE	CASES	CONTROLS	EXPOSED CONTROLS	MDRR
Crockett et al.	Ulcerative colitis	Isotretinoin	122,192	366,576	0.13%	1.27
Chou et al.	Acute pancreatitis	DPP-4 inhibitors	6,799	27,196	14.15%	1.11

Table 3. Sample size and statistical power. The minimum detectable relative risk (MDRR) was computed using the rate of exposed in the controls, and using an alpha of 0.05 and power of 0.80 (beta of 0.20).

For comparison, the original study by Crockett et al. included 4,428 cases and 21,832 controls, and reported an odds ratio of 4.36 (95% CI 1.97-9.66). The original study by Chou et al. included 1,957 cases and 7,828 controls, and reported an odds ratio of 1.04 (95% CI 0.89-1.21).

11 Strengths and Limitations of the Research Methods

Strength

- The two case-control studies that are replicated represent typical studies that are published all the time.
- The use of negative control exposures allows the evaluation of systematic error inherent to each study design.

Limitations

- Even though many potential confounders will be included in this study, there may be residual bias due to unmeasured or misspecified confounders.

12 Protection of Human Subjects

The use of the Truven Health MarketScan® Commercial Claims and Encounters Database was reviewed by the New England Institutional Review Board (IRB) and was determined to be exempt from broad IRB approval, as this research project did not involve human subjects research.

13 Management and Reporting of Adverse Events and Adverse Reactions

This study uses coded data that already exist in an electronic database. In this type of database, it is not possible to link (i.e., identify a potential causal association between) a particular product and medical event for any individual. Thus, the minimum criteria for reporting an adverse event (i.e., identifiable patient, identifiable reporter, a suspect product, and event) are not available and adverse events are not reportable as individual adverse events reports. The study results will be assessed for medically important results.

14 Plans for Disseminating and Communicating Study Results

The study results will be posted on the OHDSI website after completion of the study. At least one paper describing the study and its results will be written and submitted for publication to a peer-reviewed scientific journal.

15 Appendix 1

15.1 Negative control exposures for the Crockett study replication

EXPOSURE CONCEPT ID	EXPOSURE CONCEPT NAME
732893	Bupivacaine
1563600	Chorionic Gonadotropin
1598819	Clomiphene
981709	dorzolamide
1363053	Doxazosin
989482	Dutasteride
996416	Finasteride
1512480	Ibandronate
1502905	Insulin Glargine
1550023	Insulin Lispro
1347384	irbesartan
1386957	Labetalol

989878	Lidocaine
1102527	Meperidine
1103640	Methadone
705944	Methylphenidate
708298	Midazolam
1313200	Nadolol
1114122	Nalbuphine
717136	Neostigmine
753626	Propofol
1513103	Raloxifene
781182	ramelteon
1334456	Ramipril
965748	Scopolamine
916005	Solifenacin
924566	tamsulosin
1341238	Terazosin
902427	Timolol
913782	tolterodine
704599	Triazolam
780442	varenicline
1524674	zoledronic acid

15.2 Negative control exposures for the Chou study replication

EXPOSURE CONCEPT ID	EXPOSURE CONCEPT NAME	NESTING CONCEPT ID	NESTING NAME
1105775	Aminophylline	37203741	Bronchospasm and obstruction
924939	Bisacodyl	35702117	Gastrointestinal motility and defaecation conditions
938044	brinzolamide	35606985	Glaucoma
732893	Bupivacaine	438112	Neoplastic disease
954819	cevimeline	35606954	Sjogren's syndrome
795113	Chlorzoxazone	36516951	Back pain
1563600	Chorionic Gonadotropin	37119655	Infertility female
1350310	cilostazol	37622411	Phlebosclerosis
1517070	desmopressin	36718449	Urinary incontinence
989482	Dutasteride	37119607	Benign prostatic hyperplasia
1140088	Dyphylline	37203741	Bronchospasm and obstruction
943634	epinastine	36009773	Rhinitis allergic
757352	Eszopiclone	436962	Insomnia
19027958	fesoterodine	36718449	Urinary incontinence
1588712	Follicle Stimulating Hormone	37119655	Infertility female
1315865	fondaparinux	37622411	Phlebosclerosis
1536743	ganirelix	37119655	Infertility female

1784444	Ivermectin	36110310	Arthropod infestation
932815	Levobunolol	35606985	Glaucoma
1136422	levocetirizine	36009773	Rhinitis allergic
987366	lubiprostone	35702117	Gastrointestinal motility and defaecation conditions
1589795	Luteinizing Hormone	37119655	Infertility female
704943	Methocarbamol	36516951	Back pain
915855	olopatadine	35607032	Conjunctivitis allergic
1110942	omalizumab	37203741	Bronchospasm and obstruction
918906	oxybutynin	36718449	Urinary incontinence
922868	Permethrin	36110310	Arthropod infestation
19025115	picosulfate sodium	35702117	Gastrointestinal motility and defaecation conditions
945286	Pilocarpine	35606985	Glaucoma
40163718	prasugrel	37622411	Phlebosclerosis
951279	Prilocaine	438112	Neoplastic disease
781182	ramelteon	436962	Insomnia
1136487	ropivacaine	37522270	Surgery
965748	Scopolamine	436962	Insomnia
19012925	silodosin	37119607	Benign prostatic hyperplasia
1336926	tadalafil	37119607	Benign prostatic hyperplasia
1341238	Terazosin	37119607	Benign prostatic hyperplasia
704599	Triazolam	436962	Insomnia
1311276	vardenafil	36919202	Sexual dysfunction

16 References

1. Vandenbroucke, J.P. and N. Pearce, *Case-control studies: basic concepts.* Int J Epidemiol, 2012. **41**(5): p. 1480-9.
2. Rothman KJ, G.S., Lash TL, *Modern epidemiology. 3rd ed.* 2008., Philadelphia:: Wolters Kluwer Health/Lippincott Williams & Wilkens.
3. Madigan, D., M.J. Schuemie, and P.B. Ryan, *Empirical performance of the case-control method: lessons for developing a risk identification and analysis system.* Drug Saf, 2013. **36 Suppl 1**: p. S73-82.
4. Crockett, S.D., et al., *Isotretinoin use and the risk of inflammatory bowel disease: a case-control study.* Am J Gastroenterol, 2010. **105**(9): p. 1986-93.
5. Chou, H.C., W.W. Chen, and F.Y. Hsiao, *Acute pancreatitis in patients with type 2 diabetes mellitus treated with dipeptidyl peptidase-4 inhibitors: a population-based nested case-control study.* Drug Saf, 2014. **37**(7): p. 521-8.
6. Ryan, P.B., et al., *Empirical performance of a new user cohort method: lessons for developing a risk identification and analysis system.* Drug Saf, 2013. **36 Suppl 1**: p. S59-72.
7. Whitaker, H.J., et al., *Tutorial in biostatistics: the self-controlled case series method.* Stat Med, 2006. **25**(10): p. 1768-97.
8. Voss, E.A., et al., *Accuracy of an automated knowledge base for identifying drug adverse reactions.* J Biomed Inform, 2017. **66**: p. 72-81.

9. Winnenburg, R., et al., *Leveraging MEDLINE indexing for pharmacovigilance - Inherent limitations and mitigation strategies.* J Biomed Inform, 2015. **57**: p. 425-35.
10. Duke, J., J. Friedlin, and X. Li, *Consistency in the safety labeling of bioequivalent medications.* Pharmacoepidemiol Drug Saf, 2013. **22**(3): p. 294-301.
11. Evans, S.J., P.C. Waller, and S. Davis, *Use of proportional reporting ratios (PRRs) for signal generation from spontaneous adverse drug reaction reports.* Pharmacoepidemiol Drug Saf, 2001. **10**(6): p. 483-6.
12. Banda, J.M., et al., *A curated and standardized adverse drug event resource to accelerate drug safety research.* Sci Data, 2016. **3**: p. 160026.
13. Schuemie, M.J., et al., *Empirical confidence interval calibration for population-level effect estimation studies in observational healthcare data.* Proc Natl Acad Sci U S A, 2018. **115**(11): p. 2571-2577.
