# Supplementary material for: A plea to stop using the case‐control design in retrospective database studies
Source: Stat Med. 2019 Aug 22;38(22):4199–208. doi: 10.1002/sim.8215 (PMC6771795; doi:10.1002/sim.8215)
Supplement: Supplementary file 2 — SIM_8215‐Supp‐0002PublicationCountV02.pdf [file SIM-38-4199-s002.pdf]

Counting retrospective database studies using a case-control design in literature

PubMed query

The following PubMed query was formulated, consisting of a subquery to identify case-control studies (blue) and a subquery identifying healthcare databases (green):

"Case-Control Studies" [Mesh:NoExp] AND ((taiwan AND (NHIRD [Title/Abstract] OR (national [Title/Abstract] AND health [Title/Abstract] AND insurance [Title/Abstract] AND database [Title/Abstract]))) OR (Premier [Title/Abstract] AND database [Title/Abstract] NOT "academic search" [Title/Abstract]) OR (Solucient [Title/Abstract] AND database [Title/Abstract]) OR (Cerner Health Facts [Title/Abstract]) OR (Ingenix [Title/Abstract]) OR (LabRx [Title/Abstract]) OR (IHCIS [Title/Abstract]) OR (marketscan [Title/Abstract] or ((truven [Title/Abstract] OR thomson [Title/Abstract]) AND market [Title/Abstract])) OR (Medstat [Title/Abstract]) OR (pharmetrics [Title/Abstract]) OR (healthcore [Title/Abstract]) OR ((united healthcare [Title/Abstract] OR UnitedHealthcare [Title/Abstract]) AND database [Title/Abstract]) OR ((GPRD [Title/Abstract] OR CPRD [Title/Abstract] OR "general practice research" [Title/Abstract] OR "clinical practice research" [Title/Abstract]) AND database [Title/Abstract]) OR (Group Health [Title/Abstract] AND database [Title/Abstract]) OR (("HCUP" [Title/Abstract] OR "Healthcare Cost and Utilization Project" [Title/Abstract] OR "National Inpatient Sample" OR "Nationwide Emergency Department Sample" OR "Kids Inpatient Database") AND database [Title/Abstract]) OR (("MEPS" [Title/Abstract] OR "Medical Expenditure Panel Survey" [Title/Abstract]) AND database [Title/Abstract]) OR (NAMCS [Title/Abstract] OR "National Hospital Ambulatory Medical Care Survey" [Title/Abstract]) OR (("NHIS" [Title/Abstract] OR "National Health Interview Survey" [Title/Abstract]) AND database [Title/Abstract] NOT korea) OR ("National Health Insurance Service" [Title/Abstract] AND korea) OR (("HIRA" [Title/Abstract] OR "Health Insurance Review and Assessment" [Title/Abstract]) AND database [Title/Abstract] AND korea) OR (Kaiser [Title/Abstract] AND database [Title/Abstract]) OR (Cleveland Clinic [Title/Abstract] AND database [Title/Abstract]) OR (Lovelace [Title/Abstract] AND database [Title/Abstract]) OR (Henry Ford [Title/Abstract] AND database [Title/Abstract]) OR (("i3 Drug Safety" [Title/Abstract] OR i3 [Title/Abstract]) AND database [Title/Abstract]) OR (Geisinger [Title/Abstract] AND database [Title/Abstract]) OR (PHARMO [Title/Abstract] AND netherlands) OR ((IPCI [Title/Abstract] OR "Integrated Primary Care Information" [Title/Abstract]) AND netherlands) OR (Regenstrief [Title/Abstract] AND "medical record" [Title/Abstract]) OR ("Partners Healthcare" [Title/Abstract]) OR ("Rochester Epidemiology" [Title/Abstract]) OR ("health improvement network"[Title/Abstract]) OR ("Japan Medical Data Center" OR ("JMDC" [Title/Abstract] AND "japan")) OR (optum [Title/Abstract] AND database [Title/Abstract]) OR ("medicaid patients" [Title/Abstract] OR "medicaid beneficiaries" [Title/Abstract] OR "Medicaid Analytic eXtract") OR ("medicare patients" [Title/Abstract] OR "medicare beneficiaries" [Title/Abstract] OR ("medicare" AND ("research identifiable files" OR "research identifiable file" OR "limited dataset" OR "limited data set" OR "limited data sets") OR ("medicare claims"))) OR (("Health Maintenance

Organization" [Title/Abstract] OR "HMO Research" [Title/Abstract]) AND database [Title/Abstract]) OR ("claims data" [Title/Abstract] OR "claims database" [Title/Abstract] OR "administrative database" [Title/Abstract] OR "insurance database" [Title/Abstract]) OR ("ICD-9" [Title/Abstract] OR "international statistical classification" [Title/Abstract] OR "international classification of diseases" [Title/Abstract] OR "ICD-10"[Title/Abstract]) OR ("electronic medical record" [Title/Abstract] OR "electronic health record" [Title/Abstract] OR "computerized primary care data" [Title/Abstract]) OR ((("SEER" OR "Surveillance Epidemiology and End Results") AND "Medicare")) OR ("Intercontinental Marketing Services" OR (("IMS" OR "database") AND ("LRx" OR "Lifelink" OR "Disease Analyzer" OR "Disease Analyser" OR "LabRx" OR "Pharmetrics")) OR ("IMS" AND ("Ambulatory" OR "Oncology") AND "EMR")) OR ("explorys" [Title/Abstract]) OR ("humedica" [Title/Abstract]) OR (("Humana" OR "Comprehensive Health Insights") AND ("claims" OR "database")) OR (("blue cross blue shield" [Title/Abstract] OR "BCBS" [Title/Abstract]) AND ("claims" OR "database")) OR ("commercial health plan" AND "claims") OR ("healthcare databases" OR "population-based healthcare data") OR (National Health and Nutrition Examination Survey OR ("NHANES" AND "database")) OR ("Sweden registries") OR ("pedianet") OR ("gepard" [Title/Abstract] AND database))

Results

The query was executed on PubMed on April 26, 2019. The number of citations found per year is show in Table 1. The same information is show graphically in Figure 1. The lower counts in 2018 and 2019 are likely due to the fact that not all publication in those years have been indexed by the National Library of Medicines.

Table 1. Number of PubMed citations found per calendar year

Year	Count	Year	Count
1989	4	2005	157
1990	16	2006	147
1991	19	2007	147
1992	22	2008	191
1993	30	2009	195
1994	30	2010	210
1995	43	2011	222
1996	50	2012	280
1997	57	2013	350
1998	45	2014	351
1999	53	2015	419
2000	72	2016	434
2001	79	2017	436
2002	103	2018	240
2003	107	2019	26
2004	146		

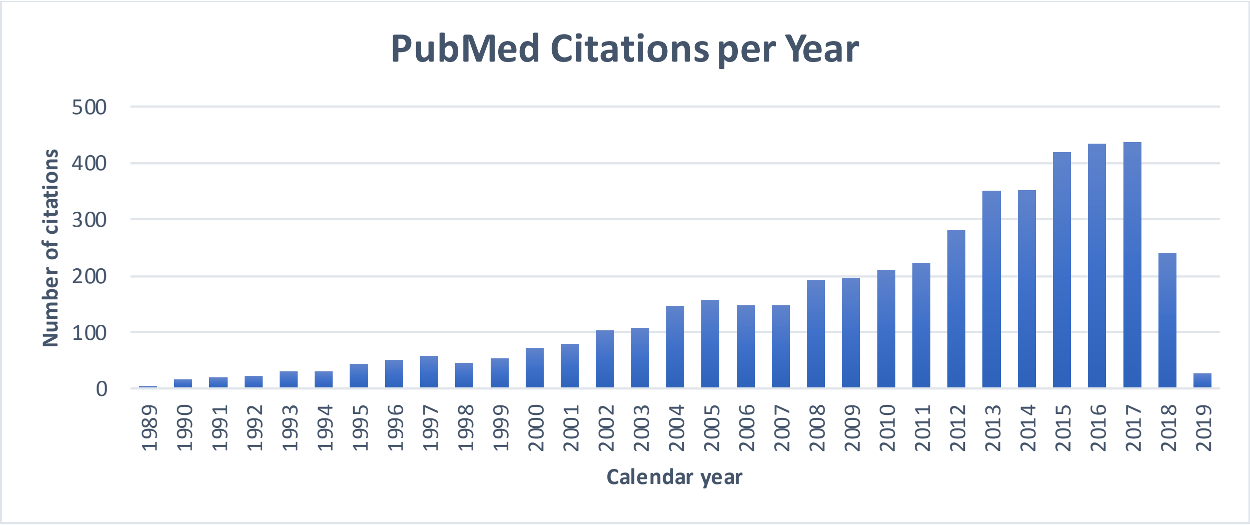

Figure 1. Number of PubMed citations found per calendar year
